# Evaluation of anti-HBs seropositivity rates in children with familial mediterranean fever

**DOI:** 10.1007/s00431-025-06221-6

**Published:** 2025-05-29

**Authors:** Sıla Atamyıldız Uçar, Eray Tunce, Serap Ata, Ebru Oğultekin Vazgeçer, Betül Sözeri

**Affiliations:** 1 Department of Pediatric Rheumatology, Umraniye Training and Research Hospital, University of Health Sciences, Istanbul, Türkiye; 2 Department of Pediatrics, Umraniye Training and Research Hospital, University of Health Sciences, Istanbul, Türkiye

**Keywords:** Anti-HBs, Colchicine, Hepatitis B virus, Familial Mediterranean Fever, Seroprotection, Vaccination

## Abstract

The aim of this study was to evaluate and compare anti-HBs seroprotection rates and antibody titers in pediatric familial Mediterranean fever (FMF) patients to healthy controls. This cross-sectional, single-center study included FMF patients followed at a tertiary pediatric rheumatology center between August 2016 and August 2024. Only patients who had received HBV vaccination according to the national vaccination schedule and had priorly documented anti-hepatitis B surface antibody (anti-HBs) titers were included. Anti-HBs levels >  = 10 mIU/mL were considered seroprotective against HBV. A healthy control group, matched by age and gender with FMF patients included for comparison. FMF patients were categorized as colchicine-resistant and colchicine-responsive FMF patients. A total of 153 FMF patients and 158 healthy controls were included. FMF patients exhibited significantly lower anti-HBs seroprotection rates (46.4% vs. 58.2%; p = 0.037) and lower median anti-HBs titers (8.5 mIU/mL [IQR, 2–49.5] vs. 20.1 mIU/mL [IQR, 2–107.5]; p = 0.013) compared to healthy controls. Among FMF patients, males showed higher seroprotection rates (n = 44, 55.7%) compared to females (n = 27, 36.5%) (p = 0.017). In the FMF group, anti-HBs seroprotection rates declined with increasing age, from 75% in the youngest cohort (< 8 years) to 41.7% in adolescents (15–18 years) (p = 0.022). The seroprotective anti-HBs rate was significantly lower in colchicine-resistant FMF patients (n = 32, 45.1%) compared to the control group (n = 92, 58.2%) (p = 0.04). None of the patients tested positive for HBsAg, and no new HBV infections developed during a median follow-up of 67 months (IQR, 36–76).  *Conclusion*: Children with FMF demonstrated lower anti-HBs titers and seroprotection rates compared to healthy controls. Colchicine resistance, older age, and female gender were associated with lower seroprotectivity. Serological screening for HBV immunity in FMF patients may help to guide individualized vaccination strategies.

**Table:** 

**What is Known:** • *Hepatitis B vaccine-induced immunity may wane over time and patients with chronic inflammatory diseases may exhibit altered vaccine responses.*
**What is New:** • *Anti-HBs seroprotection rates and antibody titers were significantly lower in children with familial Anti-HBs seroprotection rates and antibody titers were significantly lower in children with familial* • *Colchicine resistance, older age and female gender were associated with lower anti-HBs seroprotection rates among FMF patients.*

**What is Known:**

• *Hepatitis B vaccine-induced immunity may wane over time and patients with chronic inflammatory diseases may exhibit altered vaccine responses.*

**What is New:**

• *Anti-HBs seroprotection rates and antibody titers were significantly lower in children with familial Anti-HBs seroprotection rates and antibody titers were significantly lower in children with familial*

• *Colchicine resistance, older age and female gender were associated with lower anti-HBs seroprotection rates among FMF patients.*

## Introduction

Vaccination is one of the most effective, safest and cost-efficient methods to prevent life-threatening infections, including Hepatitis B virus (HBV) infection. Prior to the introduction of the HBV vaccine, HBV posed significant global health burden, causing acute and chronic hepatitis, which often progressed to cirrhosis, hepatocellular carcinoma and end-stage liver disease [1, 2]. According to World Health Organization (WHO), an estimated 254 million people globally live with chronic HBV infection [3]. Since the global implementation of HBV vaccination schedules, the incidence of HBV infection has markedly declined compared to the pre-vaccine era [4]. WHO recommended the integration of hepatitis B vaccination into their universal childhood immunization programs by 1997 [3, 5]. Our country, Türkiye, adopted this recommendation in 1998, incorporating a standard three-dose regimen (at 0, 1, and 6 months of age) into its national immunization schedule [6]. The HBV vaccine achieves immunogenicity rates of approximately 90–95% in healthy children when administered according to this schedule [7].

Despite effective vaccination strategies, certain populations, including individuals undergoing chemotherapy, or dialysis, as well as those with autoimmune diseases such as juvenile idiopathic arthritis (JIA), inflammatory bowel disease (IBD), systemic lupus erythematosus (SLE), type 1 diabetes mellitus (T1DM) and celiac disease (CD), demonstrate reduced HBV vaccine immunogenicity [1, 3, 8–12]. The reduced vaccination immunogenicity in autoimmune diseases is primarily attributed to impaired humoral responses. In contrast, systemic autoinflammatory diseases differ in their mechanisms, as they are driven by innate immune dysregulation rather than autoantibody production or antigen-specific T cell involvement [13]. Familial Mediterranean fever (FMF), the most common monogenic autoinflammatory disease in children, is characterized by recurrent, self-limiting episodes of fever, serositis, and synovitis [14–16]. This autosomal recessive disease is caused by mutations in the Mediterranean Fever (*MEFV*) gene, which encodes pyrin, a key protein in innate immunity that regulates inflammatory responses in the caspase-1 and interleukin-1β pathways by suppressing inflammasome activation [17–19].

Colchicine is the first-line treatment of FMF, effectively reducing attack frequency and severity and preventing amyloidosis. However, 5–10% of patients fail to achieve adequate symptom control with the maximum tolerated doses, necessitating biologic agent therapies, particularly anti-interleukin-1 agents [20–22]. While biologic agents are effective, they may increase the risk of susceptibility to infections [22, 23]. Given the chronic nature of the FMF and the potential for compromised immune response, assessing HBV vaccine efficacy in FMF patients before starting biologic agent therapy is important [24]. Moreover, uncontrolled subclinical inflammation associated with FMF may contribute to immune dysregulation and reduced vaccine immunogenicity, potentially leading to a progressive decline in vaccine-induced immunity over time. Despite its clinical significance, knowledge regarding HBV vaccine immunogenicity in pediatric FMF patients remains limited.

This study aimed to evaluate the seroprotective rates of hepatitis B surface antibody (anti-HBs) titers in pediatric FMF patients, compare them with healthy peers, and investigate potential factors influencing anti-HBs titers in pediatric FMF patients.

## Materials and methods

This cross-sectional, single-center study included patients diagnosed with FMF at a referral pediatric rheumatology department between August 2016 and August 2024. FMF diagnosis was established based on the Eurofever/PRINTO classification criteria and the Turkish pediatric FMF criteria [25–27]. Patients were eligible for inclusion if they had documented anti-HBs results in their medical records and had received HBV vaccination according to the national vaccination schedule (at 0, 1, and 6 months of age). FMF patients were categorized into colchicine-responsive (crs-FMF) and colchicine-resistant (cr-FMF) groups. Colchicine resistance was defined as experiencing more than one attack per month for at least six months while receiving the maximum age-appropriate colchicine dosage [21].

Data were retrospectively collected from medical records, including demographic characteristics, clinical manifestations, laboratory findings, treatment details, HBV vaccination history, and *MEFV* gene results. Laboratory data included anti-HBs titers and hepatitis B surface antigen (HBsAg) levels. FMF disease severity was retrospectively assessed using the International Severity Score for FMF (ISSF), categorized as mild (0–2 points), intermediate (3–5 points), and severe (6–10 points) [28].

In the cr-FMF group (71 patients), anti-HBs titers and HBsAg levels were measured before initiating biologic therapy. In the crs-FMF group (82 patients), HBV serology testing was performed at various time points during follow-up, either upon family request or as part of routine screening for school or internship requirements at the pediatric rheumatology outpatient clinic. The healthy control group compromised 158 healthy children who had completed the national vaccination schedule and underwent HBV serology testing for various reasons, including family requests, school or internship health screening or elective preoperative evaluations in the pediatric outpatient clinic. The three groups were matched by gender and age at the time of anti-HBs testing.

Anti-HBs titers ≥ 10 mIU/mL were considered seroprotective against HBV. Titers < 10 mIU/mL were classified as non-seroprotective, with titers between 2 and 9.9 mIU/mL considered as seropositive but non-seroprotective, and titers < 2 mIU/mL classified as seronegative. Patients with titers < 10 mIU/L received booster doses of HBV vaccine.

Exclusion criteria applied to both FMF and healthy control groups which included an incomplete HBV vaccination history, documented booster HBV vaccination before anti-HBs testing, known immunodeficiency or medical conditions other than FMF, inaccessible medical records, or age over 18 years.

### Study approval

Ethical approval was obtained from the Ethics Committee of the University of Health Sciences, Umraniye Training and Research Hospital (approval no: B.10.1.TKH.4.34.H.GP.0.01/224), and the study was conducted in accordance with the Declaration of Helsinki. Informed consent was obtained from legal guardians of all participants.

### Statistical analyses

Statistical analyses were performed by using IBM SPSS (Statistical Package for Social Sciences) Software version 29.0 (IBM Co., Armonk, NY, USA) and graphs were created by using GraphPad Prism (version 10, GraphPad Software, San Diego, CA, USA). The distribution of numerical variables was assessed with visual methods (histogram) and the Kolmogorov–Smirnov/Shapiro–Wilk tests to determine normality. Descriptive statistics were presented as medians and interquartile ranges (IQR) for non-normally and distributed variables or as means and standard deviations for normally distributed variables, while categorical variables were expressed as frequencies and compared using the chi-square test or Fisher’s exact test. Non-normally distributed numerical data were analyzed using the Mann–Whitney U test or the Kruskal–Wallis test, as appropriate. Student’s t-test was used for normally distributed variables. For statistical consistency, anti-HBs titers below the detection limit of 2 mIU/mL were assigned a value of 2 mlU/mL (n = 108, 34.7%), while titers exceeding 1000 mIU/mL were capped at 1000 mIU/mL (n = 8, 2.3%) for calculations. Scatter plot analysis was used to visualize the relationship between age and anti-HBs titers in patients with positive results across the study groups. A p-value less than 0.05 was considered statistically significant.

## Results

A total of 153 FMF patients and 158 healthy controls were included in the study. The median age at anti-HBs testing was 12.75 years (IQR 25–75 th, 9.04–15.74) in the FMF patients, with 74 (48.4%) being female. In the healthy control group, the median age was 11.6 years (IQR, 8.34–14.92 years), with 83 (52.5%) being female. There were no significant differences between FMF and the healthy control groups in terms of age at anti-HBs testing (p = 0.21) or gender distribution (p = 0.46).

### Characteristics of FMF patients

The baseline demographic and clinical characteristics of FMF patients are summarized in Table 1. The median age at symptom onset was 4 years (IQR, 2–6.8 years), while the median age at diagnosis was 5 years (IQR, 3.1–7.7 years). The median diagnosis delay was 6 months (IQR, 4.8–18 months), and the median duration of colchicine treatment before anti-HBs testing was 5.9 years (IQR, 2.9–9.1 years). The median follow-up duration was 67 months (IQR, 36–76 months). The most common FMF symptoms were abdominal pain (n = 138, 90.2%), fever (n = 137, 89.5%), arthralgia (n = 88, 57.5%), myalgia (n = 67, 43.8%), arthritis (n = 34, 22.2%) and chest pain (n = 31, 20.3%).

**Table 1:** Demographic and clinical features of FMF patients

	Total FMF (*n* = 153, 100%)	cr-FMF (*n* = 71, 46.4%)	crs-FMF (*n* = 82, 53.6%)	*p*-value*
Female	74 (48.4)	35 (49.3)	39 (47.6)	0.83
Age at symptom onset, years	4 (2–6.8)	3.5 (2–7)	4.3 (3–6.8)	0.17
Age at diagnosis, years	5 (3.1–7.7)	5 (3–8)	5.5 (3.8–7.6)	0.28
Follow-up time, months	67 (36–76)	67 (46–74)	67 (31.7–79.3)	
Duration of colchicine treatment, years	5.9 (2.9–9.1)	6.8 (3.5–10.4)	5.5 (2.6–7.9)	0.028
Annual number of attacks^χ^	12.85 ± 8.6	17.45 ± 8.9	8.65 ± 5.6	< 0.001
Parental consanguinity	44 (28.8)	17 (23.9)	27 (32.9)	0.221
Family history of FMF	93 (60.8)	42 (59.2)	51 (62.2)	0.7
*MEFV* variations				
Pathogenic homozygous	84 (54.9)	58 (81.6)	26 (31.7)	
Pathogenic compound heterozygous	28 (18.3)	13 (18.3)	15 (18.2)	
Pathogenic and uncertain compound heterozygous	6 (3.9)	-	6 (7.3)	
Pathogenic heterozygous	22 (14.3)	-	22 (26.8)	
Uncertain significance heterozygous	10 (5.4)	-	11 (13.4)	
Uncertain significance homozygous	1 (0.5)	-	1 (1.2)	
Uncertain significance compound heterozygous	1 (0.5)		1 (1.2)	
ISSF severity score	3 (0–6)	6 (5–7)	1 (0–2)	< 0.001
ISSF severity category				< 0.001
Mild (0–2 score)	74 (48.4)	-	74 (90.2)	
Intermediate (3–5 score)	36 (23.5)	28 (39.4)	8 (9.8)	
Severe (6–10 score)	43 (28.1)	43 (60.6)	-	

The data were presented as number (percentages) or median (interquartile range 25–75 percentile)

^χ^ The data was presented as mean and standard deviations

^*^p-values represent comparisons between cr-FMF and crs-FMF patient groups

*cr-FMF *colchicine-resistant FMF*, crs-FMF *colchicine-responsive FMF*, ISSF *International severity score for FMF*, n *number*, m *months*, MEFV *mediterranean fever*, y *year

The most frequent *MEFV* gene variations were pathogenic homozygous mutations (n = 84, 54.9%), with M694 V/M694 V being the most prevalent (n = 73, 47.7%), followed by compound heterozygous mutations with M680I/M694 V (n = 15, 9.8%) and M694 V/V726 A (n = 9, 5.9%). Additionally, pathogenic heterozygous M694 V (n = 11, 7.2%), pathogenic homozygous M680I/M680I (n = 9, 5.9%) mutations were identified. At the time of anti-HBs titer testing, the median disease activity score, measured by the ISSF severity score, was 3 (0–5.7). Based on ISSF classification, disease severity was categorized as mild in 48.4% (n = 74), intermediate in 23.5% (n = 36) severe in 28.1% (n = 43) (Table 1).

Among FMF patients, 71 was colchicine resistant and 82 was colchicine responsive. The characteristics and comparison of FMF subgroups are summarized in Table 1. The median age at diagnosis did not significantly differ between cr-FMF (5 years, IQR: 3–8 years) and crs-FMF (5.5 years, IQR: 3.8–7.6 years) groups (p = 0.28). The mean annual attack number was significantly higher in cr-FMF patients than in crs-FMF patients (17.45 ± 8.9 vs. 8.65 ± 5.6) (p < 0.001). Additionally, cr-FMF patients had significantly higher ISSF scores compared to crs-FMF patients (p < 0.001) (Table 1).

The most common symptoms in cr-FMF patients were fever (n = 69, 97.2%), abdominal pain (n = 69, 97.2%), arthralgia (n = 49, 69%), chest pain (n = 22, 31%) and arthritis (n = 21, 29.6%), all of which were significantly more frequent than in crs-FMF patients (fever: n = 68, 82.9%, p = 0.004; abdominal pain: n = 68, 84.1%, p = 0.007; arthralgia: n = 39, 47.6%, p = 0.007; chest pain: n = 9, 11%, p = 0.02; arthritis: n = 13, 15.9%, p = 0.042).

### Anti-HBs titer comparison between FMF and healthy control groups

The median anti-HBs titer in FMF patients was 8.5 mIU/mL (IQR, 2–49.5 mIU/mL), significantly lower than the median titer in the healthy control group (20.1 mIU/mL, IQR: 2–107.5 mIU/mL) (p = 0.013). Anti-HBs titers were categorized into seronegative (< 2 mIU/mL), seropositive but not seroprotective (2–9.9 mIU/mL), and seroprotective (≥ 10 mIU/mL) groups. A total of 82 FMF patients (53.6%) did not achieve seroprotective antibody titers. Among them, 60 (39.2%) were seronegative, and 22 (14.3%) were seropositive but non-seroprotective. The characteristics of seronegative FMF patients are summarized in Table 2. The seronegative rate was 39.2% (n = 60) in FMF patients and 31% (n = 49) in the healthy control group, though this difference was not statistically significant (p = 0.13). In both FMF and healthy control groups, the median age of seronegative cases was significantly higher than that of seroprotective cases (p = 0.016 and p < 0.001, respectively) (Table 3).

**Table 2:** Characteristics of seronegative FMF patients

	Seronegative FMF (*n* = 60, 39.2%)
Female	38 (63.3)
Age at symptom onset, years	4.5 (2.5–7)
Age at diagnosis, years	5.4 (3–8)
Age at anti-HBs testing, years	13.9 (10.9–15.8)
Follow-up time, months	67.5 (36.5–78.5)
Duration of colchicine treatment, years	7 (3.3–9.7)
Annual number of attacks^χ^	13.7 ± 9.9
Parental consanguinity	12 (20)
Family History of FMF	30 (50)
*MEFV* variations	
Pathogenic homozygous	32 (53.3)
Pathogenic compound heterozygous	11 (18)
Pathogenic and uncertain compound heterozygous	3 (5)
Pathogenic heterozygous	12 (20)
Uncertain significance heterozygous	1 (1.6)
Uncertain significance homozygous	1 (1.6)
Uncertain significance compound heterozygous	-
ISSF severity score	3 (0–5.7)
ISSF severity category	
Mild (0–2 score)	28 (46.7)
Intermediate (3–5 score)	17 (28.3)
Severe (6–10 score)	15 (25)

The data were presented as number (percentages) or median (interquartile range 25–75 percentile)

^χ^ The data was presented as mean and standard deviations

*FMF *familial mediterranean fever*, IQR *interquartile ranges*, ISSF *international severity score for FMF*, n *number*, m *months,* MEFV *mediterranean fever*, y *year

**Table 3:** Comparisons between colchicine resistant FMF patients and healthy control group

	FMF (*n* = 153)	Healthy Control (*n* = 158)	*p*-value^α^	**cr-FMF** (*n* = 71)	**crs-FMF** (*n* = 82)	*p*-value^β^
Female	74 (48.4)	83 (52.5)	0.46	35 (49.3)	39 (47.9)	0.83
Age at anti-HBs levels tested, years	12.75 (9.04–15.74)	11.6 (8.34–14.92)	0.21	12.6 (9.1–15.6)	12 (8.9–15.8)	0.16
Anti-HBs titers, mIU/mL	8.5 (2–49.5)	20.1 (2–107.5)	0.013	6 (2–67.4)	9 (2–38.1)	0.67
Seroprotected anti-HBs rates	71 (46.4)	92 (58.2)	0.037	32 (45.1)^λ^	39 (47.6)^ω^	0.75
Seroprotected anti-HBs titers, mIU/mL	53 (30.8–119)	88.5 (32.5–185.8)	0.006	72 (38.2–141.5)	39.4 (23–100)	0.055
Age at seroprotected anti-HBs titers, years	11.2 (7.9–15.7)^ϕ^	10.1 (7.5–13.9)^ε^	0.38	12.7 (7.7–16.5)	13.3 (8.2–13.3)	0.17
Seronegative anti-HBs rates	60 (39.2)	49 (31)	0.13	28 (39.4)	32 (39)	0.081
Age at seronegative anti-HBs titers, years	13.9 (10.94–15.8)^ϕ^	14.1 (11.2–16.2)^ε^	0.59	13.5 (11.1–15.6)	14.7 (10.7–16.3)	0.29

*Anti-HBs *hepatitis B surface antibody, *cr-FMF *colchicine resistant familial Mediterranean fever*, crs-FMF *colchicine responsive familial Mediterranean fever*, FMF *familial Mediterranean fever*, IQR *interquartile ranges*, n *number

The data were presented as number (percentages) or median (interquartile range 25–75 percentile)

^α^The comparison was between cr-FMF group and crs-FMF groups

^β^The comparison was between FMF group and healthy control group

^λ^p = 0.04 for the comparison between the rate of anti-HBs seroprotected cr-FMF patients (n = 32, 45.1%) with healthy control group (n = 92, 58.2%)

^ω^p = 0.075 for the comparison between the rate of anti-HBs seroprotected crs-FMF patients (n = 39, 47.6%) with healthy control group (n = 92, 58.2%)

^ϕ^p = 0.016 for the comparison between age at seroprotected anti-HBs titers and age at seronegative anti-HBs titers in FMF patients

^ε^p < 0.001 for the comparison between age at seroprotected anti-HBs titers and age at seronegative anti-HBs titers in healthy control cases

The seroprotection rate of anti-HBs was significantly higher in the healthy control group (58.2%, n = 92) compared to the FMF group (46.4%, n = 71) (p = 0.037). When comparing median seroprotective anti-HBs titers, the FMF patients had 53 mIU/mL (IQR, 30.8–119 mIU/mL), while the healthy control group had a median of 88.5 mIU/mL (IQR, 32.5–185.8 mIU/mL), with a statistically significant difference (p = 0.006) (Table 3).

A significant association was found between gender and anti-HBs seroprotection rates in FMF patients, with males demonstrating higher seroprotection rates (n = 44, 55.7%) compared to females (n = 27, 36.5%) (p = 0.017). In contrast, no significant association was observed in the healthy control group (males: n = 43, 57.3% vs. females: n = 49, 59%) (p = 0.829). When the ages of male and female FMF patients within both the seroprotective and non-seroprotective groups were compared, no statistically significant differences were observed (p = 0.455 and p = 0.851, respectively). In the seroprotective group, the median age was 11.6 years (IQR, 8.4–15.8) for males and 9.9 years (IQR, 7.6–14.9) for females. In the non-seroprotective group, the median age was 13.4 years (IQR, 11.1–15.7) for males and 13.2 years (IQR, 10.8–16.3) for females.

Among FMF patients with myalgia (n = 67, 43.8%), a significantly lower seroprotection rates (n = 25, 37.3%) was observed compared to patients without myalgia (n = 46, 53.5%) (p = 0.047). Other FMF attack symptoms were not significantly associated with anti-HBs seroprotection rates. None of the FMF patients or healthy controls tested positive for HBsAg.

Patients were grouped based on their age at anti-HBs testing into the following categories: 0–7 years, 8–11 years, 12–14 years, and 15–18 years. When stratified by age, anti-HBs seroprotection rates demonstrated a significant decline across age groups in both the FMF and healthy control groups. In the FMF group, anti-HBs seroprotection decreased from 75% in the youngest age group (0–7 years) to 41.7% in the oldest (15–18 years) (p = 0.022). A similar trend was observed in the control group, where the highest positivity rate was recorded in the youngest age group (94.7%) and the lowest in the oldest (48.7%) (p = 0.002). Across the entire cohort, anti-HBs protectivity exhibited a significant reduction with increasing age (p < 0.001). The age comparison between groups has been made and illustrated in Table 4.

**Table 4:** Comparison of anti-HBs titers in FMF and healthy controls across different age groups

	**FMF Group (153, 100%)**					**Healthy Control (158, 100%)**				
	Total *n *(%)	< 8 20 (13.1)	8–11 49 (32)	12–14 36 (23.5)	15–18 48 (31.4)	Total *n* (%)	< 8 19 (12)	8–11 66 (41.8)	12–14 34 (21.5)	15–18 39 (24.7)
Seronegative Anti-HBs rates, n (%)	60 (39.2)	5 (25)	14 (28.6)	18 (50)	23 (47.9)	49 (31)	-	18 (27.3)	13 (38.2)	18 (46.2)
Seropositive and non-seroprotected Anti-HBs rates, n (%)	22 (14.4)	-	11 (22.4)	6 (16.7)	5 (10.4)	17 (10.8)	1 (5.3)	8 (12.1)	6 (17.6)	2 (5.1)
Seroprotective Anti-HBs rates, n (%)	71 (46.4)	15 (75)	24 (49)	12 (33.3)	20 (41.7)	92 (58.2)	18 (94.7)	40 (60.6)	15 (44.1)	19 (48.7)
Anti-HBs titers, mIU/mL	8.4 (2–49.5)	34.5 (5.3–68.4)	9.5 (2–59.5)	3 (2–29.7)	3.5 (2–80.9)	20.05 (2–107.5)	111 (72.5–218)	20.5 (2–90.2)	6.5 (2–79.7)	9.5 (2–45.1)
Anti-HBs seroprotective titers, mIU/mL	56 (30–117)	39.4 (23–85.5)	59.4 (31.7–129)	34.2 (27.5–56.3)	104 (38.6–148)	88.5 (32.5–185.8)	119 (75.8–237.5)	63.1 (26.4–180.5)	97 (35.6–769)	45.1 (22–173)

*Anti-HBs* hepatitis B surface antibody, *FMF* familial Mediterranean fever, *IQR* interquartile ranges, *n* number

The diagnostic delay (p = 0.071), the total duration from the onset of symptom to the age at anti-HBs testing (p = 0.12), and annual number of attacks (p = 0.85) were not significantly associated with anti-HBs seroprotection rates. No significant differences were found in median anti-HBs titers (p = 0.7) or anti-HBs seroprotection rates (p = 0.35) across ISSF severity categories. No cases of HBV infection were detected during a median follow-up of 67 months (IQR, 36–76 months).

### Anti-HBs titer comparison between crs-FMF and cr-FMF groups

The seroprotective anti-HBs rate was significantly lower in cr-FMF group (n = 32, 45.1%) compared to the control group (n = 92, 58.2%) (p = 0.04), whereas the difference in the crs-FMF group (n = 39, 47.6%) did not reach statistical significance (p = 0.075). The comparison of cr-FMF and crs-FMF patients is presented in Table 3. The median anti-HBs titer was 6 mIU/mL (IQR, 2–67.4 mIU/mL) in cr-FMF patients and 9 mIU/mL (IQR 2–38.1 mIU/mL) in crs-FMF patients, with no significant difference between the groups (p = 0.67). The median anti-HBs titers in the healthy control group was significantly higher than in crs-FMF group (p = 0.019). Although the control group also had a higher median titer than the cr-FMF group, this difference did not reach statistical significance (p = 0.089) (Table 3).

When comparing age groups between FMF subgroups, a significant decreasing trend in anti-HBs protectivity with increasing age was observed in crs-FMF patients (p = 0.047). However, no significant age-related difference in anti-HBs seroprotectivity was found in FMF subgroup (p = 0.148) (Table 4). There were no statistically significant differences in annual attack frequency between Anti-HBs seroprotective rates in cr-FMF (p = 0.82) and crs-FMF patients (p = 0.88).

## Discussion

In this study, we found that children with FMF had significantly lower anti-HBs seroprotection rates and anti-HBs titers compared to healthy controls. Notably, older age and female gender were associated with reduced seroprotection rates among FMF patients. Furthermore, colchicine-resistant FMF patients demonstrated significantly lower anti-HBs seroprotection rates compared to healthy controls, highlighting a potential immunological vulnerability in this group considering the potential impact of more severe disease severity and chronic inflammation. This finding suggests that impaired HBV vaccine-induced immunity may be associated not only to the presence of FMF itself but also to colchicine resistance status. Overall, our findings underscore the importance of serological monitoring and individualized vaccination strategies for FMF patients, particularly those with long standing disease and higher disease burden.

Previous studies from Türkiye reported higher anti-HBs seroprotective rates in healthy children compared to our healthy control group. Large cohorts with younger mean ages reported rates of 66.4% to 69.2% [30, 31], whereas the seroprotection rate in our healthy controls was 58.2%. This discrepancy may be attributed to the older median age of our cohort. Supporting our findings, s study with a mean age of 12.77 years reported a seropositivity rate of 61.2% [32]. In literature, it has been demonstrated anti-HBs seropositivity declines progressively after ages of 7 to 15 years [30–33]. Consistent with these findings, our study also identified older age as a significant factor influencing anti-HBs seroprotective rates in both FMF and healthy children, with significantly lower seropositivity rates in those aged eight years and older.

In addition to age-related factors, we found that males had higher rates of seroprotection compared to females in our cohort. This observation led us to further explore whether age differences might account for the observed gender disparity in seroprotection rates. We compared the ages of female and male FMF patients separately within seroprotective and non-seroprotective groups. These analyses revealed no statistically significant age differences between genders in either subgroup. Therefore, the observed gender disparity in seroprotection rates appears to be independent of age. This finding may suggest that sex-related immunological differences, rather than age distribution, may contribute to variations in HBV vaccine-induced immunity among FMF patients. In our healthy control group, there was no significant difference in anti-HBs seroprotection rates between genders, which was consistent with some previous reports in the literature [30, 34]. However, in the FMF group, a higher seroprotection rate was observed in male patients, and to our knowledge this has not been previously reported. The previous studies have demonstrated higher seroprotection rates in females following HBV vaccination [35]. This unexpected finding in FMF patients from the general population warrants further investigation.

Reduced anti-HBs seroprotective titers have been reported in patients with autoimmune diseases, such as JIA, SLE, IBH, CD as well as in dialysis patients [8–12, 34, 36]. Maritsi et al. found that JIA patients had a 55% seropositivity rate, compared to 92% in healthy controls [8]. Similarly, in a study from our country, reported an anti-HBs seropositivity rates of 59.1% (n = 155) in their treatment-naive JIA patients, compared to 72.9% (n = 274) in the healthy controls [34]. Another study comparing newly diagnosed JIA patients with healthy controls found lower rates of anti-HBs titers (120.8 IU/L vs 184.9 IU/L) but no significant between the rate of anti-HBs seroprotection rates (64.7% vs 68.7%) [36]. Additionally, in a study of 351 patients with IBD and CD, 56.7% (n = 199) had non-seroprotective anti-HBs levels [37]. Our findings further support the notion that chronic autoinflammatory diseases may influence vaccine-induced immunity. In contrast to autoimmune diseases, FMF is primarily driven by innate immune dysregulation rather than autoantibody production or adaptive immune dysfunction. None of the FMF patients in our cohort had comorbidities that could independently affect anti-HBs seroprotection, reinforcing the potential role of FMF itself in modulating vaccine responses.

FMF is characterized by remitting and relapsing episodes of inflammation, typically well tolerated with colchicine therapy, cr-FMF patients require biologic therapies, particularly interleukin-1 inhibitors, to control the disease activity [38]. The presence of subclinical inflammation in uncontrolled FMF patients raises the possibility that chronic immune activation may impair vaccine-induced immunity and accelerate the decline in anti-HBs titer over time [13]. When FMF subgroups were analyzed separately, cr-FMF patients exhibited significantly lower anti-HBs seroprotection rates compared to healthy controls. This finding may reflect the greater disease burden and persistent systemic inflammation typically observed in colchicine-resistant FMF, that could potentially lead to reduced immunity. In contrast, although crs-FMF patients also showed lower seroprotection rates than healthy controls, the difference was not statistically significant. Nevertheless, the median anti-HBs titers in crs-FMF patients were significantly lower than those of healthy controls supporting the hypothesis that FMF itself-regardless of colchicine responsiveness- may contribute to reduced vaccine-induced immunity. Further studies are warranted to explore the immunological mechanisms underlying vaccine response in relation to disease severity and treatment status.

To investigate the relationship between FMF disease activity and the timing of anti-HBs testing, we assessed ISSF scores and found no significant association between ISSF severity and anti-HBs seroprotective rates. This lack of association may be attributed to the fact that all FMF patients, regardless of ISSF severity, were on long-term colchicine therapy, which may have effectively controlled subclinical inflammation and stabilized immune function. Furthermore, while ISSF is a reliable clinical tool, it may not directly capture the immunological processes underlying vaccine-induced immunity, thereby limiting its utility in predicting anti-HBs seroprotection rates. Among FMF attack-related symptoms, only myalgia demonstrated a significant association with anti-HBs seronegativity. As myalgia is a non-specific and patient-dependent symptom, its relationship with vaccine responsiveness warrants further investigation. Additionally, we also examined other potential influences, including annual attack frequency, diagnostic delay, and the duration from symptom onset to anti-HBs testing, but found no statistical differences in seroprotection rates based on these factors.

According to European Alliance of Associations for Rheumatology (EULAR) recommendations, non-live vaccines can be safely administered to pediatric patients with autoimmune inflammatory diseases receiving glucocorticoids and disease-modifying anti-rheumatic drugs with a protective immune response [24]. Given this, FMF patients with seronegative anti-HBs titers can safely receive the HBV vaccine under the supervision of primary care physicians, pediatricians or pediatric rheumatologists to maintain adequate protection against HBV infection.

As FMF symptoms often persist into adulthood, and studies in adult populations have highlighted the potential risks of HBV infection associated with chronic inflammatory conditions in patients with rheumatic diseases, the importance of comprehensive hepatitis serological screening before initiating immunosuppressive therapies recommended by international guidelines. In March 2023, the Centers for Disease Control and Prevention recommended universal HBV screening for all adults aged ≥ 18 years to enhance early detection and prevention of HBV infection [39].

As a cross-sectional study, one limitation is the lack of longitudinal data to assess changes in anti-HBs titers over time in FMF patients while correlating with disease activity. Future prospective studies are warranted to monitor anti-HBs titers, in both seropositive patients and also seronegative patients who receive booster vaccinations, to evaluate seroconversions rates. Additionally, such studies should also focus on colchicine-resistant FMF patients undergoing biologic therapy to better understand the potential relationship between disease severity and the effects of biological therapies on vaccine responses. Screening for HBV immunity could guide physicians not only prior to the initiation of immunosuppressive therapies, but also at the time of FMF diagnosis, as our findings indicate lower HBV seropositivity rates in FMF patients compared to healthy controls.

In conclusion, both the median levels of anti-HBs titers and the proportion of seroprotected FMF patients were lower than those of healthy control subjects. Colchicine resistance, older age and being female gender found to be associated with lower rates of anti-HBs seroprotectivity. While the exact mechanisms remain unclear, these findings emphasize the importance of HBV serological screening in FMF patients as a preventative measure to guide individualized vaccination strategies, particularly for those requiring immunosuppressive and biological therapies. Further research is warranted to investigate the long-term immune responses to vaccination in FMF patients and to identify factors contributing to reduced vaccine immunogenicity in this population.

## Data Availability

The datasets generated and/or analyzed during the current study are available from the corresponding author on reasonable request.

## Abbreviations

anti-HBs: Hepatitis B surface antibody
HBsAg: Hepatitis B surface antigen
HBV: Hepatitis B virus
CD: Celiac disease
cr-FMF: Colchicine-resistant familial Mediterranean fever
crs-FMF: Colchicine-responsive familial Mediterranean fever
EULAR: European Alliance of Associations for Rheumatology
FMF: Familial Mediterranean fever
IBD: Inflammatory bowel disease
ISSF: International Severity Score for FMF
JIA: Juvenile idiopathic arthritis
MEFV: Mediterranean Fever
T1DM: Type 1 diabetes mellitus
SLE: Systemic lupus erythematosus
WHO: World Health Organization
